# How to prevent and avoid barriers in co-production with family carers living in rural and remote area: an Italian case study

**DOI:** 10.1186/s40900-021-00259-0

**Published:** 2021-03-17

**Authors:** Eleonora Gheduzzi, Cristina Masella, Niccolò Morelli, Guendalina Graffigna

**Affiliations:** 1 School of Management, Politecnico di Milano, Milan, Italy; 2 Department of Psychology, EngageMinds Hub Consumer, Food & Health Engagement Research Center, Università Cattolica del Sacro Cuore (Milano), Milan, Italy

**Keywords:** Co-production, Co-destruction, Co-creation, Carer, Rural, Remote, Vulnerable, Marginalise, Fragile, Patient engagement

## Abstract

**Background:**

Co-production has been widely recognised as a potential means to reduce the dissatisfaction of citizens, the inefficacy of service providers, and conflicts in relations between the former and the latter. However, the benefits of co-production has begun to be questioned: co-production has often been taken for granted, and its effects may not be effective. To understand and prevent unsuccessful citizen and provider collaboration, the recent literature has begun to focus on the causes of co-destruction. This paper investigates how the barriers that may arise during the co-production of a new social service with family carers can be identified and interpreted.

**Methods:**

To investigate this topic, we undertook a single case study - a longitudinal project (Place4Carers (Graffigna et al., BMJ Open 10:e037570, 2020)) intended to co-produce a new social care service with and for the family carers of elderly patients living in rural and remote areas. We organised collaborative co-assessment workshops and semi-structured interviews to collect the views of family carers and service providers on the co-production process. A reflexive approach was used in the analysis for collecting the opinions of the research team that participated in the co-production process.

**Results:**

The analysis revealed four main co-production barriers: lack of trust, lack of effectiveness of engagement, participants’ inability (or impossibility) to change and the lack of a cohesive partnership among partners. Despite these findings, the project increases carers’ satisfaction, competence and trust in service providers by demonstrating the positive effects of co-production.

**Conclusions:**

Our article confirms that co-creation and co-destruction processes may coexist. The role of researchers and service providers is to prevent or remedy co-destruction effects. To this end, we suggest that in co-production projects, more time should be spent co-assessing the project before, during and after the co-production process. This approach would facilitate the adoption of adjustment actions such as creating mutual trust through conviviality among participants and fostering collaborative research between academia and organisations that are not used to working together.

## Plain English summary

The case study investigated in this paper focuses on the possible barriers that may arise during co-production between family carers of elderly people living in rural and remote areas and health and social care experts for the planning of a new service. Co-production requires a substantial contribution of citizens in designing and implementing a new service. In line with recent open debate of co-production research, our inquiry critically reflects on the experience of carer involvement. This study supports practitioners and researchers in preventing barriers before, during and after co-production with vulnerable citizens. It also illustrates possible service solutions to support family carers in looking after their elderly relatives at home, highlighting the importance of this informal category in the health and social care system.

Researchers and practitioners have adopted co-production for years by highlighting its effectiveness. However, there has been a considerable number of negative empirical examples of co-production, especially involving vulnerable citizens. Although the involvement of vulnerable citizens may be more challenging than usual, their involvement has paramount importance as their voices are rarely heard and their needs are not satisfied by existing service systems. This study is a first attempt to identify ways to prevent failure when involving vulnerable citizens.

## Introduction

In the last two decades, the involvement of patients and citizens in health and social care has become increasingly popular [1]. Among the forms of public engagement, co-production has been identified as a possible solution for several managerial issues, especially in the health and social care sector [2]. Recent studies have suggested co-production as a means to increase citizens’ satisfaction and trust in service providers [3], enhance innovation [4], and improve the effectiveness and efficiency of products and services [5–7]. Its fame has increased to such an extent that the concept of co-production has ‘enchanted’ (sic) its audience; it seems to have become a magic solution (sic) for both public and private challenges [8]. However, this optimistic view of co-production is unrealistic [9]. Organisations may encounter difficulties in involving citizens. For instance, misalignment among goals, power [10], knowledge [11], expectations, and engagement [12] may reduce the possibility of equal interaction. For this reason, scholars have started to challenge co-production by highlighting that its costs and benefits should be further investigated [13].

Unfortunately, the reasons that may inhibit co-production are not easy to identify [14]. For instance, an interactional process between citizens and providers may fail because citizens do not have enough information about the topic of discussion [15]. In contrast, it may fail because the organisation does not want to share some information with citizens [14] or because the local rules and regulations do not allow organisations to share that information [16]. Finally, the failure may be caused by all three reasons, demonstrating the complexity of this field.

Understanding how to identify and interpret barriers in co-production has both practical and theoretical benefits [7]. On the one hand, organisations may succeed in preventing or limiting barriers that can generate negative effects during co-production. On the other hand, scholars may be able to critically analyse co-production and its effects, opening the way to improvements in the application of co-production [8].

Interest in the concept of co-destruction has emerged as a possible way to explore and understand co-production barriers by looking at their negative effects on citizens, providers and other participants [17, 18]. Co-destruction was first introduced in the private service literature, which defined it as ‘an interactional process between service systems that results in a decline in at least one of the systems’ well-being (which, given the nature of a service system, can be individual or organisational)’ [19]. Despite the importance of co-destruction, the number of studies that investigate co-destruction in the health literature is quite limited [20], highlighting the urgency of further study in this field [14, 17].

On the basis of these considerations, this paper investigates the barriers that arose during the co-production of a social and community service with family carers of elderly people living in remote and rural areas and studies how they can be identified and interpreted by using co-destruction.

## Theoretical background

### Challenges involving vulnerable citizens in the health and social care field

The existing healthcare literature addresses not only the facilitators but also the potential barriers and negative effects of involving vulnerable and fragile citizens (e.g.), [1, 20]. Since barriers may arise before or during the involvement process [21], we distinguished antecedents barriers from process barriers. Then, drawing on Ludwig et al.(2020) classification, we clustered some of the main antecedents barriers, process barriers and negative effects in four groups: citizens, providers or research, teams and system [1] (Table 1). This classification helps to clarify who can limit the involvement of vulnerable and fragile citizens in the health and social care filed with an ecosystem perspective [15] at different moments (i.e. before, during the involvement process) [21]. Given the focus of this study, we highlighted in grey the barriers and effects that refer to a co-production process (see Table 1), while in white the ones that refer to other participant forms of collaboration (e.g. patient and public involvement, community engagement, patient engagement). We preferred to adopt a broad focus on the concept of investigation because co-production’s definition is still debated and not homogeneous [26].

**Table 1:** Well-documented shortcomings of public involvement and their possible negative effects in the health and social care field. In grey, the factors/outcomes revealed by the analysis of co-production processes

ANTECEDENT BARRIERS	PROCESS BARRIERS	NEGATIVE EFFECTS
***Citizens***	***Team (Citizens and Researchers/Providers)***	***Citizens***
Lack of competences [15, 20, 22–25]	Conflicts [22, 26]	Damage to ethics obligations [27]
Lack of trust [26, 28–30]	Different culture [26, 28, 29]	
Lack of training of citizens [21, 27, 31–33]	Different opinions and strong personalities [34]	Damage relationship [35]
Lack of citizens’ motivation [24, 31]	Lack of feedback exchange [34]	Additional burden on citizens [1]
Lack of healthy and cognitive condition [1]	Lack of attention to individual needs [21]	Fatigue to participate [1]
Being frail/with severe illness [1]	Tokenism [22]	Citizens felt compelled and disempowered [22]
Lack (or perceived) of power [1, 22, 23, 28, 34–36]	Usage of technical language [22]	Increasing inequalities [24]
Lack of citizens’ financial compensation [21, 37]		Low citizens’ satisfaction toward results [24]
Citizens speaking different languages [25]		
Low citizens’ awareness [24]		
***Researchers/Providers***		***Researchers/Providers***
Lack of skilled facilitator [35]		Damage research careers [35]
Lack of motivation [1, 15, 31]		Damage research credibility [35]
Lack of competences [35, 36]		Damage relationship [35]
Perceived lack of citizens’ competences [1]		Misrepresent findings [27]
		Additional fatigue [38]
***System***	***System***	***System***
Lack of resources [1, 27]	Misuse of resources [15]	Overuse/underuse of resources [15]
Lack of time [25, 29, 37]	Lack of transparency [21, 23, 34]	Damage interpersonal relationship [27]
Lack of funding [34]		Unreliable and unsatisfied results [24]
Complexity of the network [20]		Additional costs & effort [24]
Initiatives’ project related [1, 26]		
Lack of inclusion of marginalized citizens [35, 39]		
Does not clarify expectations [1, 21, 36]		
Does not clarify roles [1, 22]		
Does not clarify purpose of involvement [22]		
Mobility issues [20, 21, 28, 30, 35, 39]		

In co-production, **antecedent barriers** may predate the process itself. First, the lack of provision of the necessary information, competence and skills may make participants unable to understand providers’ speech [22], providers incapable of collaborating with other participants [35] and valuing citizens’ experiential knowledge [23, 40] and facilitators fail to manage the discussion [27]. Second, the lack of motivation of participants [15] and clarification of the purpose and roles of the process may facilitate co-production failure [22]. Third, the short and sporadic collaborations among participants with few resources and funding limit the possibility of creating trustful relationships among participants [41].

Moreover, **process barriers** may arise during the process of co-production. First, the exchange among participants may create conflicts that, if not harmonised by the facilitator, risk affecting the success of co-production [26]. Second, the usage of technical language and the underestimation of citizens’ suggestions may prevent the true involvement of citizens in decision-making processes [22].

Finally, co-production may have **negative effects** on citizens, providers/researchers and the system. In the first case, it may make citizens feel compelled [22, 42] and may risk harming their privacy and rights [27]. Second, the adoption of co-production in research may have several costs and risks for researchers given its complexity and time consumption [27]. Finally, it may require considerable time and resources without guaranteeing clear and unambiguous results [24, 27], compromising the health and/or research system.

Although the list of barriers of co-production in the health and social care literature seems quite well explored, few attempts in the literature have explained how to individuate and interpret these barriers [1]. Barriers to co-production are usually reported in broad terms [43] without highlighting the process by which negative effects are achieved. Moreover, almost all of these barriers have been identified by researchers without collecting citizens’ perspectives [43].

### Co-destruction

Co-destruction is an interactional process [19] that aims to explain how the direct or indirect interactions between participants belonging to the same service system may reduce the well-being of at least one of the parties [14]. Since co-production captures a wide variety of activities in which citizens and other service system stakeholders interact [44], it can be considered an interactional process, and the co-destruction theoretical framework can support the identification and explanation of how barriers can generate negative effects [18].

Co-destruction may arise **during** exchanges among participants and can lead to negative effects at both the individual and system levels [45]. However, recent studies have enlarged this view by considering two other possible reasons for failure. Co-destruction may occur **before** the interactional process due to the presence of pre-existing conditions that negatively influence the process [17, 46]. For instance, a lack of time or of participants’ motivation may reduce the success of the co-production process [15, 26]. Moreover, co-destruction may arise **after** the interactional process from the perceptions that participants create outside the direct interactions with providers and/or other stakeholders [17, 47]. For instance, inadequate treatment/service may lead to a negative perception of patients and their carer after the interaction with clinicians.

Given the scarce health literature investigating co-destruction [20], to understand this concept, we should examine the private and public service literature. Two authors have investigated co-destruction empirically: Jarvi et al. [17] and Engen et al. [14].

Jarvi et al. (2018) [17] studied the factors that facilitate the failure of interactions between users and (public and private) providers (i.e., co-destruction) and when they emerge in business-to-consumer, business-to-business, business-to-government and government-to-consumer markets. Jarvi et al. (2018) [17] did so by considering the service provider’s perspective in the private and public sectors. Through this analysis, they identified eight causes of co-destruction: *absence of information, insufficient level of trust, lack of clear expectations, inability to serve, inability to change, mistakes, customer misbehaviour*, and *blaming* (sic) that can occur before, during and after the collaboration process. The first cause, i.e., the ‘***absence of information’***, arises from the inability of providers and users to understand and share information. The second cause, i.e., ‘***insufficient level of trust’***, occurs when participants are unable to believe each other. The third cause, i.e., ‘***lack of clear expectation’***, is determined by users’ inability to express or to conceptualize their expectations clearly. The fourth cause, the ‘***inability to serve’,*** arises from the incapacity of providers to achieve users’ expectations effectively and on time. The fifth cause, i.e., ‘***inability to change’***, refers to the incapacity of both providers and users to modify their behaviour and approaches according to new environments or contingencies. The sixth cause, i.e., ‘***mistakes’***, arises from unintended events such as the application of incorrect assumptions or the purchase of incorrect products. The seventh cause, i.e., ‘***customer misbehaviour’***, refers to the misuse of resources or the negative behaviour of users that has negative impacts on the service/project. Finally, the last cause, i.e., ‘***blaming’***, arises from users complaining about products or services [17]. Thus, this research clarifies that barriers may arise at different times and may be continuous over time.

Jarvi et al. (2018) study gives some original insights but it does not include users’ perspective in identifying the reasons of interactional process’ failure [14] and it uses negative and nonconstructive terminologies. Nevertheless, it is a pioneering research on co-destruction in the public service field and, thus, it can be a useful guideline for analysing critically co-production process.

Engen et al. (2020) investigated the causes of co-destruction in the public service literature by studying the direct interactions between service users and the Social Insurance Agency and the Tax Agency in Sweden [14]. Unlike Jarvi et al. (2018), the causes were investigated using both citizens’ and providers’ perspectives. Their research identified four reasons for co-destruction: inability to serve, mistakes, lack of bureaucratic skills, and lack of transparency. The first two causes of co-destruction are aligned with the framework identified by Jarvi et al. in 2018. The other two reasons for co-destruction arise from the adoption of a broader perspective that includes service users and third parties. This demonstrates the importance of investigating the causes of failure by including the perspectives of all participants in the service network. Moreover, this novel research clarifies the link between barriers and their effects. Co-destruction may change over time, moving from co-creation to co-destruction of value, and its effects may have different impacts, from value diminution to irreparable loss [14, 46].

### Data and methods

To investigate the barriers to the co-production process, we used a case study methodology, which facilitates the understanding of current phenomena before, during and after co-production, making the phenomena inseparable from the context [48, 49]. More precisely, we investigated the barriers to co-production by using co-destruction as the theoretical lens to understand and reveal them. The adoption of this particular lens of analysis overcomes at least two existing limitations of the health and social care literature. First, as a process, it highlights when barriers arise and how they generate negative effects [17]. Second, from a system view, it collects the perspectives of all participants of the networks, including citizens, providers and researchers.

### Case and context description

Since the purpose of our research was to investigate the causes of a specific process, we chose a case for which we had good access to the data [50]. We decided to investigate a project (called Place4Carers) in which we were involved directly as project partners. This research enabled us to reflect critically on the achievements of the project by considering the barriers encountered during its implementation. The project investigated is a longitudinal project launched to co-produce a new social and community service for the family carers of elderly citizens that use a home care service and live in a remote and rural valley in northern Italy, Vallecamonica. The aim of the project was to support family carers in their daily care of elders and improve their well-being through the launch of a new service that was co-designed by family carers, local service provider and researchers. The project is being conducted by the Università Cattolica del Sacro Cuore, a local home care agency ‘Azienda Territoriale per i Servizi alla Persona’ (ATSP), Politecnico di Milano University and the Need Institute, and it is funded by Fondazione Cariplo. Four local assisted living facilities collaborate with this project by promoting the project to the family carers, whose elders were using their home care services [51].

This research is part of a larger study intended to make a substantial contribution to the debate on the involvement of vulnerable citizens in co-production activities [52]. This research was performed in the latest phase of the study, in which we considered the implementation of the project to similar remote and rural areas. To reflect on the lessons learned during the development of the overall project, we adopted a distinctive perspective that helped us to identify the possible barriers arising from the co-production of the new social and community service with family carers, ATSP and researchers of the project team. In the co-production process, family carers were involved as users, while ATSP and researchers as providers of the new service. Moreover, given the fragility of family carers caused by medium-high level of stress and a high number of hours of caregiving [52] and the limited accessibility of local health and social care facilities in remote and rural areas, we can consider this case study a useful example of how to investigate co-production with vulnerable citizens.

During the project, ATSP and researchers involved the family carers of older patients residing in Vallecamonica in the co-design of a new public service for them. On the basis of the results from co-design workshops, the project team envisaged the new public service as comprising four activities: a training programme, mutual help meetings, citizens’ committees, and project and service information. The training programme was a set of practical courses for family carers to increase their knowledge about caring elders. The peer-to-peer meetings were groups of family carers coordinated by a psychologist, during which family carers shared their feelings and fears with each other reducing their sense of loneliness and isolation from the rest of the society. The citizens’ committee was a group of family carers, researchers and ATSP representatives set up to support the implementation of the pilot. This group met every 4 month to collect feedbacks about the service’s achievement and identify possible improvements. The project and social and community care services’ information consisted of online and offline channels (i.e., Facebook page, project website, brochure) created by the project team to spread awareness of the project and local services for the elderly. The new service was designed according to carers’ suggestions; researchers organised and summarised the service proposals that had arisen during the co-design workshops and discussed their economic and organizational feasibility with ATSP representatives. Indeed, some co-design service ideas were not feasible because the project was limited in funding, resources and time.

Through this new service, the project team expected to reach, satisfy and support family carers in their daily care of elders and to establish the service as a common practice in the local territory over time. More precisely, it expected to reach at least one third of the overall family carers eligible for the study (who were around 320 carers) [51]; to deliver every month a training course and a peer-to-peer meeting during the 18 months of the service pilot; to achieve a medium-high satisfaction rate of the training courses and peer-to-peer meetings (above 75%); to guarantee an medium-high understanding of the training courses’ content (above 75%); and to make the citizens’ committee a self-organized group of family carers and service providers that collaborate for leading and improving the new service periodically and autonomously over time.

The table below summarises the quantitative indicators used to obtain a preliminary overview of the new social care service. To assess the effectiveness of

- *the training programme*, we collected the participation rate, the level of understanding of the content (by asking participants before and after each meeting to answer three questions about the content of the course) [53] and the level of satisfaction with the course (by asking participants at the end of each course to complete a satisfaction survey of 13 items used 3-point scale of de Lima) [54, 55];
- *peer-to-peer meetings*, we collected data on the participation and satisfaction (4-point scale of Psicoclinica [56]) of family carers with regard to this activity ([57];
- *the citizens’ committee*, at the beginning of the service pilot, we self-defined, together with the ATSP, six expected outcomes from this activity (i.e. being spokesperson of family carers of Vallecamonica; encouraging interactions among carers and local service providers; defining the organizational structure of the committee; establishing one or more committee’s representative; organizing and setting committee’s activities and purposes; opening a communication channel for facilitating the exchange of messages among committee’s participants), and at the end of the pilot, we jointly checked their realisation;
- *the project and service’s information*, we ascertained knowledge about the project and the local health and social care services by checking how many new patients of the ATSP had been informed through the project’s channels. Moreover, we integrated the analysis by collecting the level of usage of the online project channels [58] (Table 2).

**Table 2:** Assessment factors

Activities	Assessment Factors	Results
***Training programme***	Number of courses	5 courses *(+ 1 cancelled)*
	Average number of participants per meeting	7 carers *(overall: 36 carers)*
	Average % of understanding by participants of course contents	88% *(31 out of 36 carers answered)*
	Average % satisfaction of participants	98% *(32 out of 36 carers answered)*
***Peer-to-peer meetings***	Number of meetings	5 meetings *(+ 3 cancelled)*
	Number of participants per meeting	6 carers *(overall: 28 carers)*
	Satisfaction of participants	−4 out of 7 carers that answered were completely satisfied (100%); −3 out of 7 carers that answered were satisfied (75%);
***Citizen committee***	Number of meetings	3 meetings *(+ 1 cancelled)*
	Average number of participants per meeting	7 carers *(overall: 22 carers)*
	Achievement of the six pre-defined goals	−3 out of 6 achievements were partially reached; − 3 out of 6 achievements were unfulfilled;
***Project and services’ information***	Number of “likes” on the project’s Facebook page	59 likes
	Number of visits to the project website	130 visualizations
	Number of downloads of informative materials on the project website	11 downloads
	Number of new patients of ATSP informed through the project’s channels (2 months’ time period)	0

Although, on average, satisfaction with the pilot was high (i.e., above 85% on average), the number of meetings organised (13 meetings) and the number of carers involved (86 carers) was quite low. The access to and use of the online channels (i.e. Facebook page, project website) created to inform the local community about the project and the local services also seemed unsatisfactory. To achieve a satisfactory number of activities and participants, the project team had to extend the pilot by 2 months to help the ATSP, which was in charge of implementing the pilot, in organising additional service activities. The context of the study may explain some of these findings, i.e., a remote and rural valley. The logistic difficulties [59] typical of this context might have influenced the participation rate. The successful and unsuccessful results reveal that the interactional process among participants generated both co-creation and co-destruction processes. On the one hand, the project increased citizens’ well-being by enhancing family carers’ satisfaction in course’s activities. However, it failed to increase the well-being of the project team because the time and resources invested was substantial considering there was a small number of family carers reached in the pilot study.

On the basis of these considerations, we deem this project suitable for investigating our research questions for three main reasons. First, it reflects on the adoption of co-production with vulnerable citizens in the health and social care sector. Second, the time horizon of analysis is medium-long, facilitating the evaluation of co-production activities during execution and beyond as suggested by co-destruction framework of Jarvi et al. Third, the involvement of family carers in the co-designed service yields both positive and negative effects, making the investigation of the barriers that arose during co-production interesting and important.

### Data collection

Following Engen’s approach, we used a variety of methods for data collection by including all the participants of the co-production network [14]: ATSP representatives, family carers and researchers that had participated to the co-design workshops and had experienced the new service. To understand the opinion of the ATSP, we used *semi-structured interviews* with three ATSP representatives responsible for implementation of the new service. To collect the perspectives of family carers, we organised two co-assessment *workshops* with ATSP representatives, researchers and family carers to discuss and evaluate together the achievements of the project [60]. Throughout the analysis, we adopted the *reflexivity approach* suggested by Bradbury et al. (2020) to gather our points of view as researchers [61] (Table 3).

**Table 3:** Data inventory

***Participants involved in the co-production activities***	***ATSP representatives***	***Family carers***	***Project researchers***
***Methods of data collection***	*Semi-structured interviews*	*Co-assessment workshops*	*Reflexivity approach*
***Number of participants***	4 representatives (out of 4)	2 workshops (one at the middle and one at the end of the pilot) involving: • 11 carers (out of 26); • 2 researchers (out of 8); • 3 ATSP representatives (out of 3).	4 researchers (out of 7)
***Participants’ characteristics***	2 Male; 2 Female	• Carers: 1 Male; 10 Female; • Researchers: 1 Male; 1 Female; • ATSP representatives: 1 Male; 2 Female.	1 Male; 3 Female

The interviews and the co-assessment workshops were designed to evaluate the co-design process, the co-delivery of the service, and the collaboration among research team members. The **interview** structure was organised as follows. First, we looked at personal involvement in the project, duties, expectations and a general evaluation of the process. Second, we used the dimensions suggested by Jarvi [17] as a guideline to check their reliability and suitability for the difficulties encountered in the project. Third, we asked for an evaluation of the collaboration among research team members. In parallel, we conducted the same process in **workshops** with carers, asking for an exhaustive evaluation of the co-production process, pros and cons of being involved in the co-design of the new social and community service, an evaluation of the new service that had been delivered and collaboration with researchers and providers. The evaluation process involved all the research team members who were effectively involved in the co-production process and in delivery of the service. All the carers were asked to participate in the focus groups, but many could not participate due to their caring duties. To the best of our knowledge, no carers refused because they were opposed to the project outcomes or process. Finally, as part of the research group, we adopted the developmental **reflexivity approach** suggested by Bradbury et al. (2020), including reflections on co-destruction, to provide suggestions on what we had learned in this project [61]. The authors of this paper were all involved in the analysis and critical reflection. Despite we did not involve an independent researcher, the authors had a specific methodological background in qualitative research ensuring the ethical and professional standards for reliable qualitative research and critical thinking. Bradbury et al. suggested involving researchers in this process to provide a personal and self-critical stance on their role [61]. The evaluation component of the project, like the entire research protocol, was approved by the ethics board of Politecnico di Milano University and the Catholic University of the Sacred Heart, Milan. Data were collected by two members of the research team: a post-doc in sociology with specific skills in qualitative research and a PhD student with expertise in service management and evaluation.

### Data analysis

The interviews took place in January 2020 in Breno (Brescia, Italy) in Vallecamonica and lasted 201 min overall, with an average of 51 min each. One interview was conducted by telephone and was the same duration as the face-to-face interviews. The co-assessment workshops took place in July and December 2019 and lasted 138 min overall. The interviews and the workshops were analysed using a deductive and then inductive approach [62]: workshops were analysed by referring to Jarvi et al. theoretical framework of co-destruction, then, by investigating new unexplored barriers of co-production. Each interview and workshop was audio-recorded with the participants’ consent and analysed by investigating their perspective on the co-production outcomes and the pros and cons of co-design. We analysed the perspectives of all participants in the service network who were involved both directly and indirectly in service delivery, as suggested by Engen et al. (2020). In particular, we enriched the analysis by investigating collaboration and the possible difficulties in communication or role identification within the research group, with family carers and with the other stakeholders of the service network. The compilation of this paper followed the Standards for Reporting Qualitative Research guidelines [63]. NM and EG, who conducted the data collection, were supervised by CM and GG to maximise the reflexivity and transparency of the process.

### Findings

The analyses of the interview and workshop data led us to identify four barriers to co-production related to trust and engagement, barriers to change and the importance of a cohesive partnership.

### The importance of trust

Lack of trust emerged as a powerful initial obstacle to co-production that influenced many refusals to participate in the first stage of the project. Both carers and research team members affirmed that in the context of Vallecamonica, it is still difficult to speak about personal problems and to ask for help from both friends and local institutions. This dimension emerged in multiple forms: towards the institution (ATSP) and towards the project team.

*“It is typical behaviour of this valley: people participate [in a new activity] only if they know [who the organiser is] or they have received the information by word of mouth” (Research team member, male, 1).*

Although the ATSP had great difficulties in promoting participation in the project, the provision of clear explanations, constant contact and interest in the carers’ experiences encouraged carers to participate and maintain their contribution to the project.

*“The first time, I was doubtful. What did they want from me? It was the first time. I was afraid that I would have to pay. But when I met [ …*.] *of the ATSP, I changed my mind. He explained the project to me, my role, and I was really happy to participate, even if I wasn’t sure how I could actually help with the project” (Carer, female, 8).*

During the first co-assessment workshop that took place during the pilot scheme, carers identified insufficient external information and communication about the project as a barrier to the project’s effectiveness. They declared that many social workers and general practitioners were not informed about the project.

*“I usually go to the support group for carers of patients with dementia [at the hospital], and they didn’t know about the project. I think it is important to connect different initiatives that all together can reach all carers” (Carer, female, 9).*

After that claim, members of the ATSP went to practitioners’ conferences in the valley and informed the coordinators of social workers. However, during the second co-assessment workshop, carers still reported that information was not widespread.

Therefore, lack of trust certainly influenced the participation of carers in the initial phase, but the positive relationship established with the ATSP and research team members convinced carers to participate in the entire project because they were positively impressed by the role that the ATSP was assigning them.

Carers who had not been convinced did not participate in any of the organised appointments. In this case, neither the ATSP nor university researchers managed to reduce the initial mistrust of carers towards strangers (as university researchers, ATSP representatives and other family carers), negatively influencing the effects of co-production.

### The importance of effective engagement

The results from our analysis of the interviews and workshops reveal difficulties in establishing effective engagement. In particular, carers who participated felt truly involved in the co-production, but in some cases, the research team made decisions without asking them for their opinion, creating friction. For example, the research team decided to postpone some education/training and support events due to the expected low participation of carers. The decision was made to avoid that trainers “*come for only a few people, considering that all of them came for free” (Research team member, male, 1).* However, carers contested this decision by stating, *“Even if there is low participation, we have to start with something. It is important, for otherwise we’ll never get started. I absolutely understand the reasons why you cancelled some meetings, and I was not angry but sorry because I need these moments and I would have preferred few participants but maybe the possibility to speak, get some relief” (Carer, female, 4).*

This claim highlights that carers felt insufficiently involved in the decision and asked for explanations. In this case, the relationship between providers and carers established in the co-production prevented this mistake from becoming a cause of co-destruction. Since this problem emerged during the first co-assessment workshop, which took place in an initial phase of service delivery, we were able to adjust the decision-making mechanism.

It also shows that although there were misunderstandings, the climate within the co-productive team was good because everyone felt at ease in explaining what they believed was wrong and required explanation and, more importantly, they were aware of the importance of participation in the project.

### Barriers to change

According to Jarvi et al. (2018), a significant barrier to successful co-production that could lead to co-destruction is the inability, of both carers and providers, to change [17]. Interviewees revealed that carers find it difficult to leave patients alone for four main reasons. First, carers usually cannot leave patients alone at home, so they must find a substitute who is both professionally trained and accepted by the patient. Second, carers usually feel responsible for and engaged in caring activities and any new possible substitute need to demonstrate that can be trusted. Third, the distinctive culture of Vallecamonica often encourages citizens to hide their family’s problems, which might be taken as signifying a personal weakness. Fourth, the ATSP as a service provider was unable to offer additional home services for giving to family carer more free time and encouraging their participation.

*“Leave him (patient) alone at home? It’s not possible, and also when the professional carer comes or the social worker, if I go away he starts to scream and cry” (Carer, female, 5).*

*“I understand you, and I also do not feel comfortable; my professional carer is not able to manage the feeding tube, and so I am always worried” (Carer, male, 10).*

*“I would like to find a professional carer to have some relief and to participate in these events, but it is very expensive” (Carer, female, 2).*

Is this, using Jarvi’s terminology, an impossibility to change or an inability to change? It is likely both. Family carers accepted to be substituted only by professionals (e.g. social workers or nurses) that they can trust and felt comfortable with. Moreover, it was not possible to provide additional hours of home care services from the local service providers (i.e. ATSP and the four local assisted living facilities) for carers involved in the project’s activities because this would have required additional human and economic resources that were not available.

Finally, carers suggested using local television broadcast to disseminate information about the project; this was done, although in a weak format (some interviews and short news items in local newspapers). As stated by the ATSP, the fees required for iterative publications and investments in marketing campaigns were particularly expensive. Since fees were not foreseen in the project budget, the project team was unable to meet the requirement.

*“I was a little bit disappointed by local journalists because they asked for a fee like it was a normal commercial spot. This is a free service to our people!” (Research team member, male, 1).*

### The strengths and weaknesses of partnership

One of the innovative features of this project was a strong partnership with two universities and the local services provider. To identify its strengths and weaknesses, it is important to include the perspectives of all participants: carers, ATSP representatives and researchers.

Carers were enthusiastic about the partnership. They felt at ease with someone who, for the first time, listened to them. Moreover, even in co-production, carers gained indirect benefits because they could speak with peers who were experiencing the same difficulties and had direct access to more information.

*“When I came here the first time I felt alone and did not know what to do. After hearing other people with the same troubles and some good suggestions, I felt more empowered” (Carer, female, 8).*

*“Having the possibility to give advice, suggestions and ideas was great even if not easy because it was difficult to find time to participate, but it was the first time that I took some time for myself. Also, having universities was something strange, but it helped us greatly to give ideas” (Carer, male, 10).*

*“Understanding the point of view of carers helps us to identify their needs better; you receive more attention. At the same time, it helped us understand what kind of doubts they had about existing services” (Research team member, female, 2).*

*“I felt very surprised and grateful for this[…]. Usually, there is not much interaction with service providers, so it was an important new opportunity” (Carer, male, 13).*

Unlike interviewees who expressed great enthusiasm for the cooperation and sense of belonging created by the project, these interviewees evidenced difficulties in collaboration and coordination within the research team. First, the ATSP complained of a lack of clarification about the team’s roles and coordination.

*“There is a difference in work style between universities and local service providers. Universities are more flexible, giving more autonomy to partners to achieve their results. We (the local home care agency) need more supervision, someone that clearly states what we have to do and at what times” (Research team member, female, 1).*

Reflecting on these criticisms, researchers admitted that universities usually give full autonomy to each coordinator of a work package; close supervision would be an act of intrusion or lack of trust by the other partners.

Second, the meeting style had an impact on the discussion of problems and ways to manage difficulties.

*“We (the ATSP) are not used to making rapid Skype or conference calls. I was not comfortable in explaining difficulties and problems about the piloting” (Research member, male, 1).*

*“We usually have a weekly meeting, not long, but just to share news and difficulties within each project. We missed that part. We need constant feedback (Research member, female, 1).*

Different organisational cultures led to this difficulty that unfortunately created less cohesion within the research group [64] and caused misunderstandings in the co-production process.

## Conclusions

This paper has reflected on the shortcomings of the co-production process when dealing with vulnerable, marginalised communities. It has done so with an empirical analysis of a path-breaking project that aimed to co-produce a support service for vulnerable family carers living in the remote and rural context of Vallecamonica. More precisely, we adopted the theoretical framework of co-destruction as a lens for identifying and interpreting barriers.

We identified four main barriers that arose before, during and after the co-production process. First, the dimension of trust emerged as a powerful barrier in engaging carers before, during and after the co-production process. Throughout the projects, the participation rate was unsatisfactory due to the “distrustful culture” [65] typical of rural areas [66]. However, carers who overcame their initial mistrust experienced positive outcomes from the service delivered. Second, the lack of effective engagement of family carers may negatively affect them. However, our research showed that the early and rapid identification of this barrier and the presence of trustful relationships among parties reduced its impact. Third, the inability to change both carers and providers may negatively influence the effects of co-production. However, participants may sometimes be unable to change because it is impossible for them to do so. In this case, it is the duty of researchers or providers to check the feasibility of services and their activities and to prevent impracticable solutions. Fourth, universities and providers may have different organisational cultures, which can generate incoherent strategies and practices in co-production regimes. However, our experience showed that different organisational cultures can be mitigated by clarifying team members’ roles and boundaries and by sharing methodologies and tools of co-production with the entire research group (not only among members who are already knowledgeable about engagement and co-production).

Despite these considerations, it would be ungenerous to say that we experienced co-destruction. Carers who decided to start participating in the project were very pleased with their active role in the new service, and the assessment of service activities revealed that they were useful and interesting for participants (e.g., the rates of satisfaction with and understanding of course content).

**Fig. 1:**
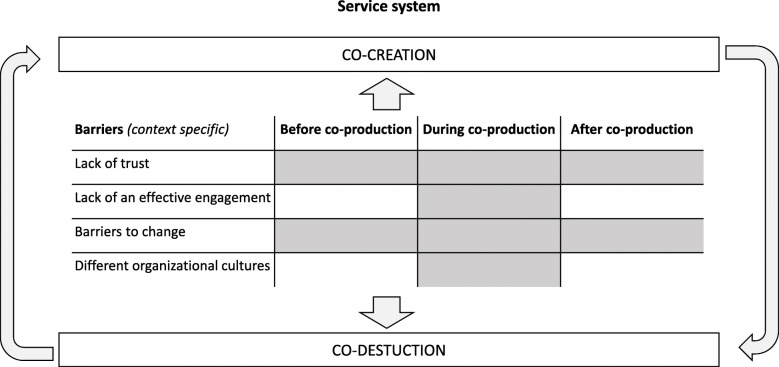
Co-destruction and co-creation in social care service co-production by adopting a service system perspective (adapted from: [14, 17])

This research confirms public and private studies by highlighting that the two dynamic processes of co-creation and co-destruction may coexist [16] (Fig. 1). Thus, co-destruction is a temporary status that may change over time and in terms of the intensity of its effect [14, 46, 67]. For instance, the lack of effective engagement of family carers would have led to more absolute co-destruction if providers and researchers had continued to adopt the same unfair decision-making mechanism over time.

Researchers and providers have the duty to limit and prevent co-destruction by adopting co-assessment methods for preventing, identifying and adjusting any possible barriers arising before, during and after co-production. To this end, it is important to collect the perspectives of all the participants of the service system involved in co-production. In particular, including citizens’ perspectives would enhance the understanding of co-production process and prevent its failure.

This research revealed new barriers and demonstrated existing ones, highlighting that barriers may vary from context to context. However, the innovativeness of this research does not refer to the barriers per se but to the approach for supporting researchers and providers in detaching and preventing barriers before, during and after the co-production process.

There is a need for more empirical studies in remote and challenging scenarios and with vulnerable populations to identify better solutions for critical issues. Moreover, it is important to strengthen a beneficial link between universities and providers to create greater effectiveness towards and with vulnerable people. In this case, it could be particularly important to foster funding for research projects aimed at collaboration between these two groups of participants.

We identified some powerful limitations in our research. First, we were not able to reach carers who participated in initial workshops but later did not attend events. It was particularly difficult to access carers who had abandoned the project due to their isolation and reluctance to speak with institutions and universities. We do not know whether they did not participate due to a lack of interest, a lack of time, or the death of the patient. On our side, we properly informed every carer about the importance of participating in every workshop for the assessment of the project. We believe that providers and all participants involved with carers should spend more time building trust relationships with carers and patients. This would have the beneficial effects of greater familiarity, the ability to provide better advice, and a greater ability to speak about needs and requirements to foster a more efficient health and social care system.

Second, to assess possible barriers to co-production, we interviewed carers twice, but research team members interviewed carers only once. In the first round of interviews/workshops, carers had a crucial role in modifying and rethinking some services. It is likely that an intermediate round of interviews with research team members would have highlighted prior problems in creating a cohesive partnership. We believe that it would be better to devise an assessment plan for co-production at different stages of the co-production process and involving all participants. Moreover, the evaluation of a collaborative research project is strongly recommended, not only at its end but also as part of a successful research team culture. In particular, specific co-assessment of the cohesive partnership should become a widely used tool in these projects. Only in this way can the barriers to co-production be uncovered to prevent co-destruction.

## Data Availability

Not applicable.

## Abbreviations

ATSP: Azienda Territoriale per i Servizi alla Persona (local home care agency)
